# New insight into the bark beetle *ips typographus* bacteriome reveals unexplored diversity potentially beneficial to the host

**DOI:** 10.1186/s40793-023-00510-z

**Published:** 2023-06-09

**Authors:** Ezequiel Peral-Aranega, Zaki Saati-Santamaría, Miguel Ayuso-Calles, Martin Kostovčík, Tereza Veselská, Karel Švec, Raúl Rivas, Miroslav Kolařik, Paula García-Fraile

**Affiliations:** 1 Microbiology and Genetics Department, Universidad de Salamanca, Salamanca, 37007 Spain; 2 Institute for Agribiotechnology Research (CIALE), Salamanca, 37185 Spain; 3 Institute of Microbiology of the Czech Academy of Sciences, Prague, 142 20 Czech Republic; 4 Associated Research Unit of Plant-Microorganism Interaction, Universidad de Salamanca-IRNASA-CSIC, Salamanca, 37008 Spain

**Keywords:** Microbial ecology, Host-microbe interactions, Insect microbiome, Lignocellulolytic enzymes, Symbionts

## Abstract

**Background:**

*Ips typographus* (European spruce bark beetle) is the most destructive pest of spruce forests in Europe. As for other animals, it has been proposed that the microbiome plays important roles in the biology of bark beetles. About the bacteriome, there still are many uncertainties regarding the taxonomical composition, insect-bacteriome interactions, and their potential roles in the beetle ecology. Here, we aim to deep into the ecological functions and taxonomical composition of *I. typographus* associated bacteria.

**Results:**

We assessed the metabolic potential of a collection of isolates obtained from different life stages of *I. typographus* beetles. All strains showed the capacity to hydrolyse one or more complex polysaccharides into simpler molecules, which may provide an additional carbon source to its host. Also, 83.9% of the strains isolated showed antagonistic effect against one or more entomopathogenic fungi, which could assist the beetle in its fight against this pathogenic threat. Using culture-dependent and -independent techniques, we present a taxonomical analysis of the bacteriome associated with the *I. typographus* beetle during its different life stages. We have observed an evolution of its bacteriome, which is diverse at the larval phase, substantially diminished in pupae, greater in the teneral adult phase, and similar to that of the larval stage in mature adults. Our results suggest that taxa belonging to the *Erwiniaceae* family, and the *Pseudoxanthomonas* and *Pseudomonas* genera, as well as an undescribed genus within the *Enterobactereaceae* family, are part of the core microbiome and may perform vital roles in maintaining beetle fitness.

**Conclusion:**

Our results indicate that isolates within the bacteriome of *I. typographus* beetle have the metabolic potential to increase beetle fitness by proving additional and assimilable carbon sources for the beetle, and by antagonizing fungi entomopathogens. Furthermore, we observed that isolates from adult beetles are more likely to have these capacities but those obtained from larvae showed strongest antifungal activity. Our taxonomical analysis showed that *Erwinia typographi*, *Pseudomonas bohemica*, and *Pseudomonas typographi* species along with *Pseudoxanthomonas* genus, and putative new taxa belonging to the *Erwiniaceae* and *Enterobacterales* group are repeatedly present within the bacteriome of *I. typographus* beetles, indicating that these species might be part of the core microbiome. In addition to *Pseudomonas* and *Erwinia* group, *Staphylococcus*, *Acinetobacter*, *Curtobacterium, Streptomyces*, and *Bacillus* genera seem to also have interesting metabolic capacities but are present in a lower frequency. Future studies involving bacterial-insect interactions or analysing other potential roles would provide more insights into the bacteriome capacity to be beneficial to the beetle.

**Supplementary Information:**

The online version contains supplementary material available at 10.1186/s40793-023-00510-z.

## Background

The European bark beetle *Ips typographus* is considered a secondary pest as it mainly attacks weakened or dying spruce conifers (*Picea abies*), thus, playing a role in the forest recycling process [1]. Nowadays, drought is severely affecting Norway spruce woodlands, one of the most important in the European forest economy, not only by directly increasing mortality rates but also weakening them, making these trees more susceptible to beetle attacks [2, 3]. Climate change also increases temperatures which in turn heighten the frequency of *I.* typographus outbreaks, leading to pheromone-mediated mass attacks, even on healthy trees. Consequently, this situation is causing the devastation of these environments [4].

Most of the *I. typographus* lifespan occurs inside the inner bark of its host, where the insect undergoes different life stages: egg, larvae, pupae, teneral adult, and adult. Log trees present a competitive environment, in which essential nutrients such as phosphorus, nitrogen, and other biomolecules (e.g. vitamin B) are scarce [5–7]. Furthermore, other beetles (*Cleridae*), parasite wasps, nematodes, and entomopathogenic fungi, amongst others, are natural enemies of *I. typographus* beetles [8–10].

Alternatively, it has been reported that some microorganisms are beneficial, or have the potential to be beneficial, to the beetles’ fitness by alleviating possible threats [1, 11–14] Although, fungal biodiversity and roles of fungi in beetle ecology have already been extensively described [13, 15, 16], there are still many uncertainties regarding its bacteriome, such as taxonomical composition, insect-bacteriome interactions, and their potential roles in beetle ecology [1].

Some studies have provided insights in the *I. typographus* bacteriome using massive amplicon sequencing [17–19]. They found that the main phylum present was *Proteobacteria*. Concretely, *Erwinia, Pseudoxanthomonas, Pseudomonas, Acinetobacter*, and *Serratia* were amongst the most abundant genera in these studies. Strains isolated from *I. typographus* beetles have already been published, although these studies covered one to three isolates [20–22].

Additionally, it has been reported that the beetle bacteriome may have the metabolic capacity to alleviate nutritional needs by the hydrolyzation of complex sugar polymers that are non-digestible to the beetle into simpler, more assimilable sugars [1]. Similarly, bark beetle-associated bacteria may have a protective role against pathogens through the synthesis of metabolites such as antibiotics, siderophores, and chitinases amongst others [23–25]. Hydrolyzation of complex sugars and antagonism to other microbes have been previously supported by the results of *in silico* and in vitro assays analysing specific strains originating from the inner part of *Ips* beetles [20, 21, 26, 27]. Also, an *in silico* approach predicted the genomic potential of different *Ips* bacteriomes [17]. All these findings support the hypothesis that the bacteriome is important for beetle fitness. Although, an extensive collection of isolates, obtained from different life stages and using different culture approaches, might provide a better understanding of the cultivable bacteriome and their metabolic potential.

In this study, we aimed to unveil potential ecological roles of *I. typographus* associated bacteria and to identify key taxa. To do that, we evaluated the capacity of these bacterial isolates to hydrolyse complex polysaccharides into simpler molecules, which may provide an additional carbon source to the *I. typographus* beetle. Our experiments also evaluated the isolates for their ability to antagonize fungi entomopathogens, which could potentially aid in protecting the beetle. Additionally, through culture-dependent and -independent techniques, we present a taxonomical analysis of the bacteriome associated with the *I. typographus* beetle during its different life stages. Taken together, our results suggest that some taxa within this bacteriome may have vital roles in maintaining beetle fitness and are ubiquitously present in their ecology.

## Materials and methods

### Sampling

We collected spruce tree logs infected with *I. typographus* in Nižbor forest (Czechia, 49°59’09.9"N 13°56’47.5"E, 390 m.a.s.l.) at two different times: January and June 2019. The sampling site is situated in a continuously forested area belonging to Protected Landscape Area Křivoklátsko. The average annual temperature is 9ºC and the average annual precipitation is 494.9 mm (Lány observatory, Czech Hydrometeorological Institute). Both times the infested logs were transported to the Institute of Microbiology of the CAS (Prague) and incubated there in the exterior in a shaded place simulating the original forest site and successively sampled for various bark beetle life stages regardless of the gender.

### Bacterial isolation

For bacterial isolation, we collected and pooled 10 *I. typographus* individuals of each developmental stage separately: adults, teneral adults, pupae, and larvae, which were then surface sterilized for 1 min with HgCl_2_ (2%) and rinsed with distilled water in aseptic conditions. Afterward, the insects were crushed, and serial dilutions were made. Finally, solutions at different concentrations (10^− 3^ to 10^− 6^) were spread onto different culture mediums: tryptic soy agar (TSA; Sigma-Aldrich, Darmstadt, Germany); M9 minimal medium (M9; K_2_HPO_4_ 0.3%, KH_2_PO_4_ 0.3%, MgSO_4_ + 7H_2_O 0.15%, CaCl_2_ + 2H_2_O 0.05%, NaCl 0.1%, NH_4_NO_3_ 0.1%, Mannitol 10% and 2% agar); nutrient agar (NA; Sigma-Aldrich, Darmstadt, Germany); and on Mitsuhashi and Maramorosch Insect modified medium (MMI; CaCl_2_·2H_2_O0.19 g/L, MgCl_2_ 46,9 mg/L, KCl 0.2 g/L, NaCl 7 g/L, NaH_2_PO_4_ 0.1739 g/L, D(+) Glucose 4 g/L, lactalbumin hydrolysate 6.5 g/L, yeast extract 5 g/L, NaHCO_3_ 0.12 g/L, pH was adjusted to 6.3–6.9 and 50 mL/L of 10% horse red blood cells (after autoclave)). The plates containing the latter culture medium were incubated at 26.5 °C with 5% O_2_ until colonies were observed. Also, AN and M9 mediums were adjusted to a final pH of 5 or 7 and cultured them at 15ºC or 26.5 ºC. Those isolates obtained in February 2019 from adult beetles were called CA, from teneral adults CYA, from Pupae CP, and larvae C1L, C2L, and C3L. Meanwhile, those adults obtained from samples of June 2019 were called AC, but if from larvae: MMI and LC when from larvae. Pure cultures were stored in 20% glycerol at − 80 °C for long-term storage.

### DNA extraction and bacterial strains identification

For bacterial strain identification, the REDExtract-N-Amp™ Tissue PCR Kit Protocol (Sigma-Aldrich, Darmstadt, Germany) was used to extract DNA as indicated by the manufacturer. The 16 S rRNA gene was amplified as described in Rivas et al., 2007 [28]. The PCR products were visualized on a 1% agarose gel after electrophoresis (60 V for 120 min), purified using the GeneJET Gel Extraction and a DNA Cleanup Micro Kit (Thermo Scientific^™^, Göteborg, Sweden), and sequenced by Sanger at Macrogen Ltd. (Madrid, Spain). The sequence fragments obtained were aligned using BioEdit [29] and the resulting sequences were compared against type strains available at the EzTaxon 16 S rRNA database using the EzBioCloud platform [30].

### Amplicon sequencing

We sampled and pooled 15 individuals of each developmental stage: adults, larvae, pupae and teneral adults, from trunks collected in June. These pooled samples were surface sterilized by subsequent washing with 70% ethanol, 2% Tween 80 (Avantor, USA) and sterile distilled water before DNA extraction. We adopted the phenol-chloroform protocol [31] for DNA extraction. Briefly, the total DNA was extracted using the phenol-chloroform–isoamyl alcohol (25:24:1) premixed solution (Sigma-Aldrich, St. Louis, Missouri, USA). The initial homogenization was done on FastPrep-24™ 5G Instrument (Irvine, California, USA) using wolf-ram beads in combination with glass beads (BioSpec Products, Inc., Bartlesville USA). DNA yield was quantified on Qubit 2.0 Fluorometer (Thermo Fisher Scientific, Waltham, Massachusetts, USA) using Qubit dsDNA BR Assay Kit and DNA quality was checked on Nanodrop (NanoDrop™ 2000c, Thermo Fisher scientific).

PCR amplification was done by kit KAPA HiFi HotStart ReadyMix (Roche, Basel, Switzerland) using an input DNA concentration of 50 ng/µl. We used labelled pairs of primer 799F 5’-AACMGGATTAGATACCCKG-3’ [32] and 1115R 5′-AGGGTTGCGCTCGTTRC-3′ [33] for 16 S marker V5-V6 region for the bacterial identification. This region was selected as these primers do not match beetle 18 S and mitochondrial 16 S rRNA gene sequences [34]. Each DNA sample was amplified in triplicates in separate 96-microtiter plates, which were subsequently pooled into one sample. Each 96-microtiter plate also included three negative controls (PCR grade water used as a template) and one positive control (a random DNA sample of one of our pure bacterial cultures was used as a template). Amplicons were then purified from oligonucleotides by SAP-Exo kit (Jena Bioscience GmbH, Germany). 1 µg of purified amplicon served as a template for library construction using KAPA HyperPlus Kit in combination with KAPA UDI primer mixes (Kapa Biosystems, Massachusetts, USA). Amplicon’s size selection of the final libraries was done by KAPA Pure Beads (Kapa Biosystems) and its effectiveness was checked on 1% agarose gel (SeaKem® LE Agarose, Lonza Group Ltd, Basel Switzerland). The amplicons size was around 300 bp. The quality of the ligated library was quantified using the EliZyme Library Quantification Kit (Elisabeth Pharmacon, Brno, Czechia). Library sequencing was done on the Illumina MiSeq platform (San Diego, California, USA) on a 2 × 300 bp paired-end reads run performed at CEITEC institute (Brno, Czechia).

### Amplicon analysis

Sequencing data were processed using QIIME 2.0 2021.8 [35]. Raw reads were demultiplexed and quality filtered using the q2-demux plugin. Afterwards, reads were denoised using the DADA2 algorithm [36] and a feature table with counts of amplicon sequence variants (ASVs) per sample was produced. Taxonomy was assigned using the q2‐feature‐classifier classify-sklearn [37] using a trained naïve Bayes classifier to assign taxa from the EzTaxon database (version 1.5). Rarefaction analysis of final ASV tables was performed to assess the completeness of the dataset and the admissible data resampling level for statistical analysis. Diversity metrics and taxonomic classification were also carried out in QIIME2. Amplicon sequences were deposited at the NCBI’s GenBank database under the Bioproject PRJNA888454.

Comparative analysis and tree construction of the metabarcoding sequences and the 16 S rRNA sequences of the isolates was performed on MEGA X software [38], which were based on the ClustalW all nucleotides alignment [39, 40]. The initial tree for the heuristic search was obtained automatically by applying Neighbor-Joining and BioNJ algorithms to a matrix of pairwise distances estimated using the Maximum Composite Likelihood (MCL) and the phylogenetic trees were generated following the Maximum Likelihood method and Tamura-Nei model [41]. Type strain sequences were collected from the EzTaxon database.

### Lignocellulose-related activity

To evaluate the capacity of the strains to produce hydrolytic enzymes of wood compounds, the in vitro hydrolysis of cellulose, xylan, starch, and pectin was tested in Petri dishes with the corresponding substrate added to TSA medium as previously reported [42, 43]. Carboxymethyl cellulose (CMC) (Sigma-Aldrich, Darmstadt, Germany) at 0.2% was added into the medium as a source of non-soluble cellulose for assessing cellulase activity. Beechwood xylan (Sigma-Aldrich, Darmstadt, Germany), potato starch (Sigma-Aldrich, Darmstadt, Germany), and citrus peel pectin (Sigma-Aldrich, Darmstadt, Germany) at a concentration of 1% were respectively added to the media for assessing xylanase, amylase, and pectinase activity. Five-microlitre drops of a bacterial suspension at McFarland 7 standard concentration were inoculated onto the surface of the plates. After incubating for a week at 28 °C, all colonies were carefully washed using sterile water. The CMC and xylan plates were stained using a 0.1% Congo Red (Panreac Química SLU, Barcelona, Spain) solution for 30 min, while the starch- and pectin-containing plates were stained using the same method but with Lugol’s solution (Panreac Química SLU, Barcelona, Spain). Three 15-minute washes of a NaCl (1 M) solution were performed for removing the excess dye.

### Antimicrobial potential

Siderophore production was evaluated on modified M9-CAS-agar medium plates as described by Jiménez-Gómez et al. [44]. The plates, prepared as previously indicated, were inoculated with 7 µL of bacterial suspension and kept at 28 °C for a week. The formation of an orange halo around the colonies was considered a positive result.

The inhibition of fungi *Lecanicillium muscarium* CCF 3297, *Beauveria brongniartii* CCF 1547, *Metarhizium anisopliae* CCF 0966, *Beauveria bassiana* CCF 5554, *Lecanicillium muscarium* CCF 6041, *Isaria farinosa* CCF 4808, *Beauveria bassiana* CCF 4422, and *Isaria fumosorosea* CCF 4401 was tested. These fungal strains were selected because they are potentially pathogenic to *Ips* beetles [9, 45, 46]. To do so, the inhibition tests were performed by streaking bacteria on TSA plates and left to grow for 5 days, at which time fungi plugs were placed next to the bacteria. Then, plates were again incubated until the fungi in controls without bacteria had growth and growth rates were compared.

## Results

### Analysis of the diversity of ***Ips typographus-***associated bacteria throughout the life cycle

Our analysis of the amplicon sequences of the 16 S rRNA V5-V6 hypervariable region obtained at different life stages (larvae, pupae, teneral adult, and adult) of *I. typographus* samples showed a relatively wide and changing bacterial diversity, strongly dominated by the phylum Proteobacteria.

We assessed alpha diversity by comparing the Shannon indices; according to our results, the bacterial diversity was higher in adults (3.6) and larvae (3.5) samples than in pupae (2.1) or teneral adults (2.8). Rarefaction curves indicated that we obtained enough sequencing depth to cover the sample diversity (Supplementary Fig. 1).

**Fig. 1:**
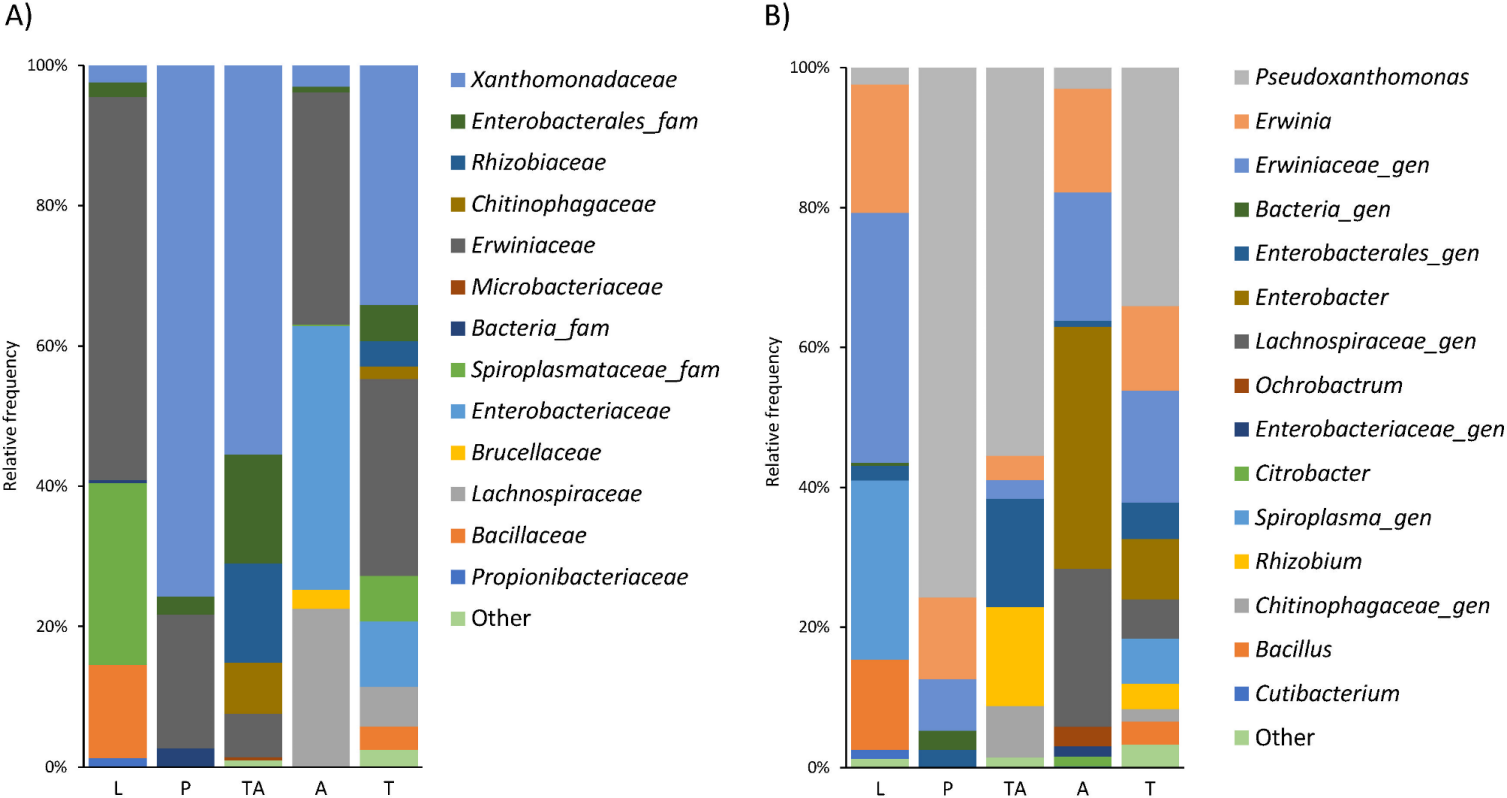
Top taxa distribution over the different life stages. Only those taxa that represent 1% or more of the total reads have been included. (A) Family level. (B) Genus level. Larval (L), pupae (P), teneral adult (TA), and adult (A). (T) Mean values amongst all samples

**Fig. 2:**
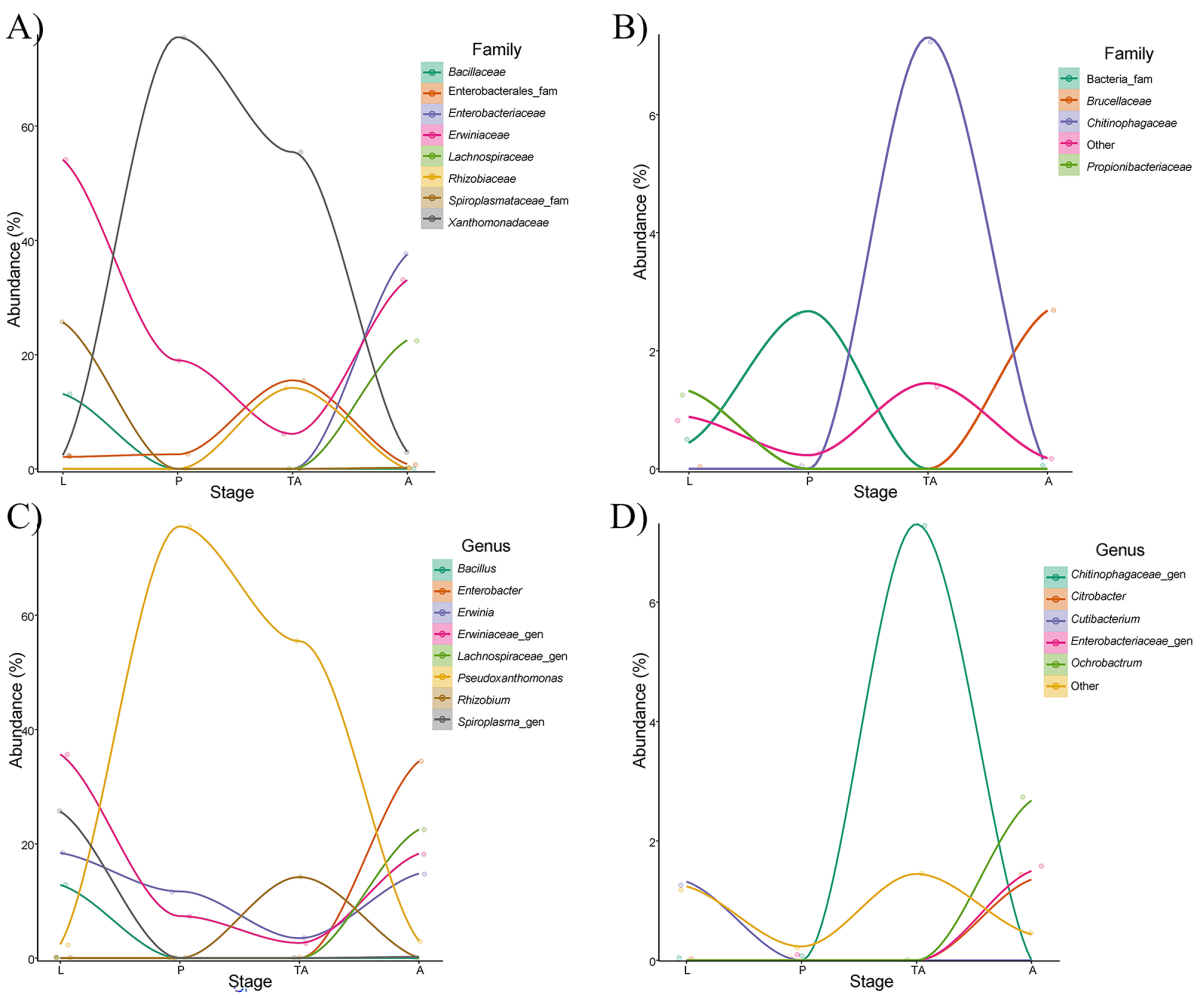
Taxa abundancy fluctuation over the different life stages. Only those taxa that represent 1% or more of the total reads have been included. (A) and (B) Family level, (C) and (D) Genus level. Larval (L), pupae (P), teneral adult (TA), and adult (A)

We identified amplicons from the phyla Tenericutes and Firmicutes in larvae and adults. Actinobacteria were found in larvae and teneral adults, and Bacteroidetes were only found in teneral adults. When we analysed diversity across the different life stages (considering taxa represented by 1% or more of the total reads) (Figs. 1 and 2) we observed that in pupae and teneral adults diversity seemed to be strongly dominated by the genus *Pseudoxanthomonas*: 75% and 55.5% of the reads, respectively. This genus represented less than 3% of the reads in larvae and adults. In larvae, the main genus was a putative new *Erwinaceae* genus (35.7%) which is also present in pupae (7.3%), teneral adult (2.6%), and adults (18.3%). The *Erwinia* genus is also ubiquitously present in all life stages (18.4%, 11.7%, 2.5%, and 14.8%, respectively). *Bacillus* and *Cutibacterium* genera were only found in larvae, with abundance percentages of 12.8% and 1.3%, respectively. Also, a putative *Spiroplasma* genus appears in larvae, with a 25.7% abundance, and it is absent in other beetle life stages but adults (just with 0,2% abundance). In pupae we detected a putative new bacterial family (2.7%) that was also found in larvae (0.4%). Regarding teneral adults, two groups were only found in this stage: *Rhizobium* (14.2%) and a putative *Chitinophagaceae* genus (7.3%). Although, the second most represented taxon was a putative *Enterobacterales* genus (15.5%) that was also present in the other stages (2.1%, 2.5%, and 0.8%, respectively). Finally, in adults we found *Enterobacter* (34.5%), a putative *Lachnospiraceae* genus (22.5%), *Ochrobactrum* (2.7%), and *Citrobacter* (1.4%) that are not present in the other stages.

### Cultured-based analysis of the diversity of ***Ips typographus-***associated bacteria

We obtained 123 bacterial isolates from different life stages of the bark beetle *I. typographus*, which were identified and classified according to the life cycle stage in which they were isolated, phylum, family, and genus (Supplementary Table 1). The isolates were included within the Proteobacteria (Pseudomonadota) (7 families), Firmicutes (Bacillota) (5 families), and Actinobacteria (Actinomycetota) (5 families) phyla. The results showed that the lowest degree of diversity at the family level was detected in the teneral adult (3 families) and pupal (5 families) stages, whereas isolates from the larval stage belonged to a total of 16 different families.

**Fig. 3:**
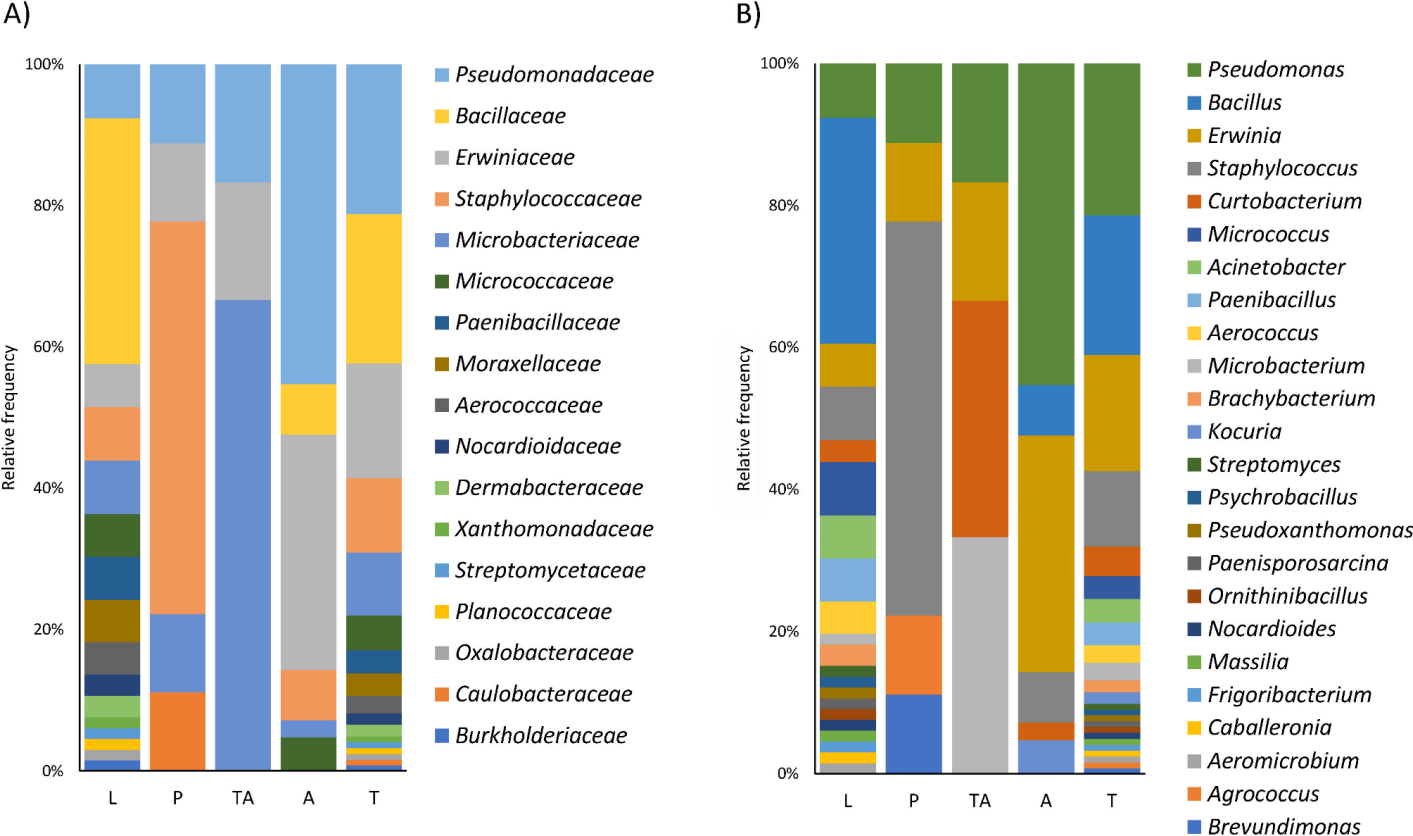
Taxonomic distribution of the isolates. 16 S rRNA sequences were used to identify and cluster isolates over different taxonomic levels and life stages. (A) family and (B) genus. Larvae (L), pupae (P), teneral adult (TA), and adult (A). Also, bars summarizing all the sequences are included (T)

Similarly, at the genus level we found the highest diversity at the larval stage, followed by the adult stage. By contrast, in the pupal and teneral adult stages, diversity was at its lowest. *Pseudomonas* and *Erwinia* were found in all the life stages of *I. typographus*; meanwhile, *Staphylococcus* and *Curtobacterium* were found in all life stages except in teneral adults and larvae, respectively. On the contrary, *Bacillus* was only detected in larvae and adults, *Microbacterium* in larvae and teneral adults, *Kocuria* in adults, and *Brevundimonas* and *Agrococcus* were only isolated from pupae. We also isolated *Aeromicrobium, Caballeronia, Frigoribacterium, Massilia, Nocardioides, Ornithinibacillus, Paenisporosarcina, Pseudoxanthomonas, Psychrobacillus, Streptomyces, Aerococcus, Paenibacillus, Acinetobacter, and Micrococcus* specifically from larvae (Fig. 3, Supplementary Table 1).

### Comparative of taxa distribution: massive amplicon sequencing vs. culture collection

When we compared the diversity obtained from both the amplicon sequence variants (ASVs) and the isolates’ 16 S rRNA gene sequence analyses, we observed that the *Erwiniaceae* family was one of the most abundant throughout the different life stages. However, this was not the same for *Xanthomonadaceae*, which was the other most abundant family according to the ASV analysis, but for which we only isolated one strain (larvae stage). Conversely, we detected *Pseudomonas* in all life stages, according to the isolates sequence analysis but in the case of the ASV analysis, *Pseudomonas* was in all stages but the larval stage (≈ 0.2% of total abundancy). Similarly, we isolated *Bacillus* in larvae and adults, but only found ASVs belonging to this genus in larvae. We isolated *Curtobacterium* strains in larval, teneral adult, and adult phases, but we only found them in teneral adults and adults ASVs. Meanwhile we isolated *Staphylococcus* in all stages, except teneral adults, but their ASVs were only found in larval and adult samples. Each of these genera represented about 0.1% of total ASVs abundance.

Considering the most representative genera obtained in the culture-independent approach and the abundance of isolates obtained in the cultures, we decided to evaluate (i) The evolution of these taxa along the different phases of the beetle’s lifespan and (ii) the similarity among ASVs and sequences obtained from *Erwinia, Bacillus, Pseudomonas, Curtobacterium, Pseudoxanthomonas*, and *Staphylococcus* isolates.

We observed that *Pseudoxanthomonas* genus was dominant in pupae and teneral adults, but barely present in larvae and adults. Conversely, *Erwinia* and the potentially new *Erwiniaceae* taxon are dominant in larvae and adult phase meanwhile their presence is lower in the intermediate stages. *Bacillus* was only found in larvae (Supplementary Fig. 2A). We found *Staphylococcus* in larvae and adults; *Pseudomonas* in all stages but larvae; and *Curtobacterium* in teneral adults and adults (Supplementary Fig. 2B).

**Fig. 4:**
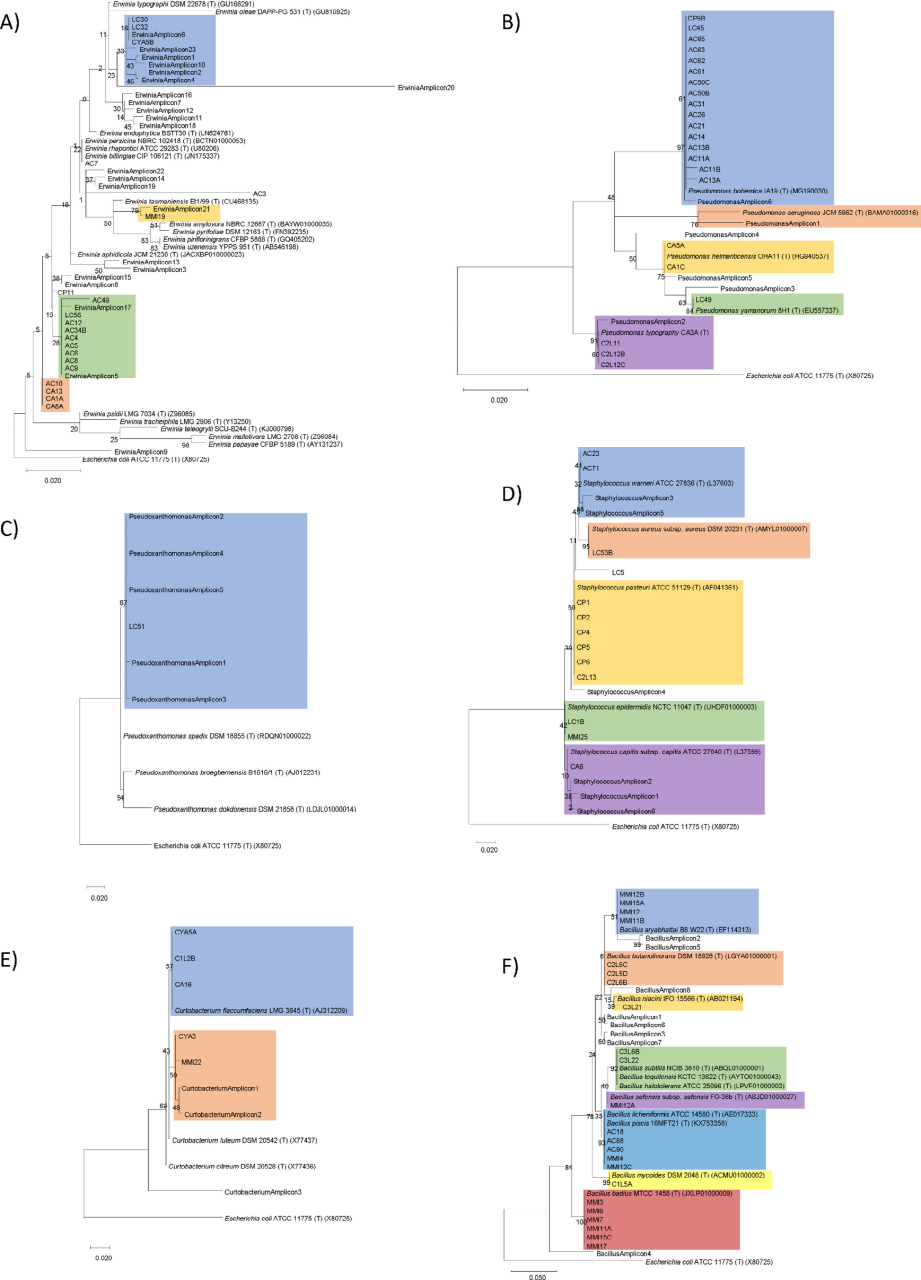
Phylogenetic tree representing distances among V5-V6 hypervariable regions of 16 S rRNA sequences from isolates of this study and ASVs identified as *Erwinia, Pseudomonas, Pseudoxanthomonas, Staphylococcus, Curtobacterium*, and *Bacillus*, respectively. Also, in each tree, closely related type strains and *Escherichia coli* genus type strain sequences are included to provide phylogenetic context. Coloured zones include group of sequences close or separated to the type strains of the genus

Considering the importance (abundance) of these genera within some life stages, we performed a phylogenetic analysis on each of these taxa together with type strains to evaluate the taxonomic placement of the isolates and the ASVs (Fig. 4).

In the *Erwinia* group phylogenetic tree (Fig. 4A and Supplementary Fig. 3) we found 3 isolates and 6 ASVs grouped close to *Erwinia typographi* DSM 22,678^T^ and an isolate and an ASV close to *Erwinia tasmaniensis* Et1/99^T^. The remaining isolates, but two, were grouped in two clades and no other type strain of the genus clusters with them. One of the clades included 2 ASVs. As for the rest of the ASVs, most were grouped in 4 independent clades with no type strains of validated species of the genus.

To further explore the phylogeny of our isolates, we calculated similarity level and constructed a phylogenetic tree including the whole 16 S rRNA sequences from the isolates of this work identified as *Erwinia* and those of the type strains of the validated *Erwinia* species. In this tree we observed 3 isolates sequences grouped with *E. typographi* DSM 22,678^T^ and one next to *Erwinia billingiae* CIP 106,121^T^; in both cases, the similarity levels among our sequences and the sequences of the type strains are above 99.3%, suggesting that these isolates can be identified within those two species. However, the remaining isolates were grouped into four different clades, 2 of them with single isolates, and no type strains of validly published species appear within these clades; moreover, the similarity levels between the sequences of these isolates and those of type strains of the genus are ≤ 98.4%; whereas the similarity among the sequences of some of the type strains of different species are higher (Supplementary Figs. 4 and 5).

In the *Pseudomonas* tree, the sequences of one isolate from larva, one isolate from pupa, 14 isolates from mature adults, and an ASV sequence are in the same clade as *Pseudomonas bohemica* IA19^T^ (Fig. 4B). Similarly, in the case of *Staphylococcus*, 2 ASVs and the sequences of two isolates from adults are grouped with *Staphylococcus warneri* ATCC 27,836^T^ (Fig. 4D).

### Assessment of bacteria isolated from ***I. typographus to*** hydrolyse bark complex molecules

**Fig. 5:**
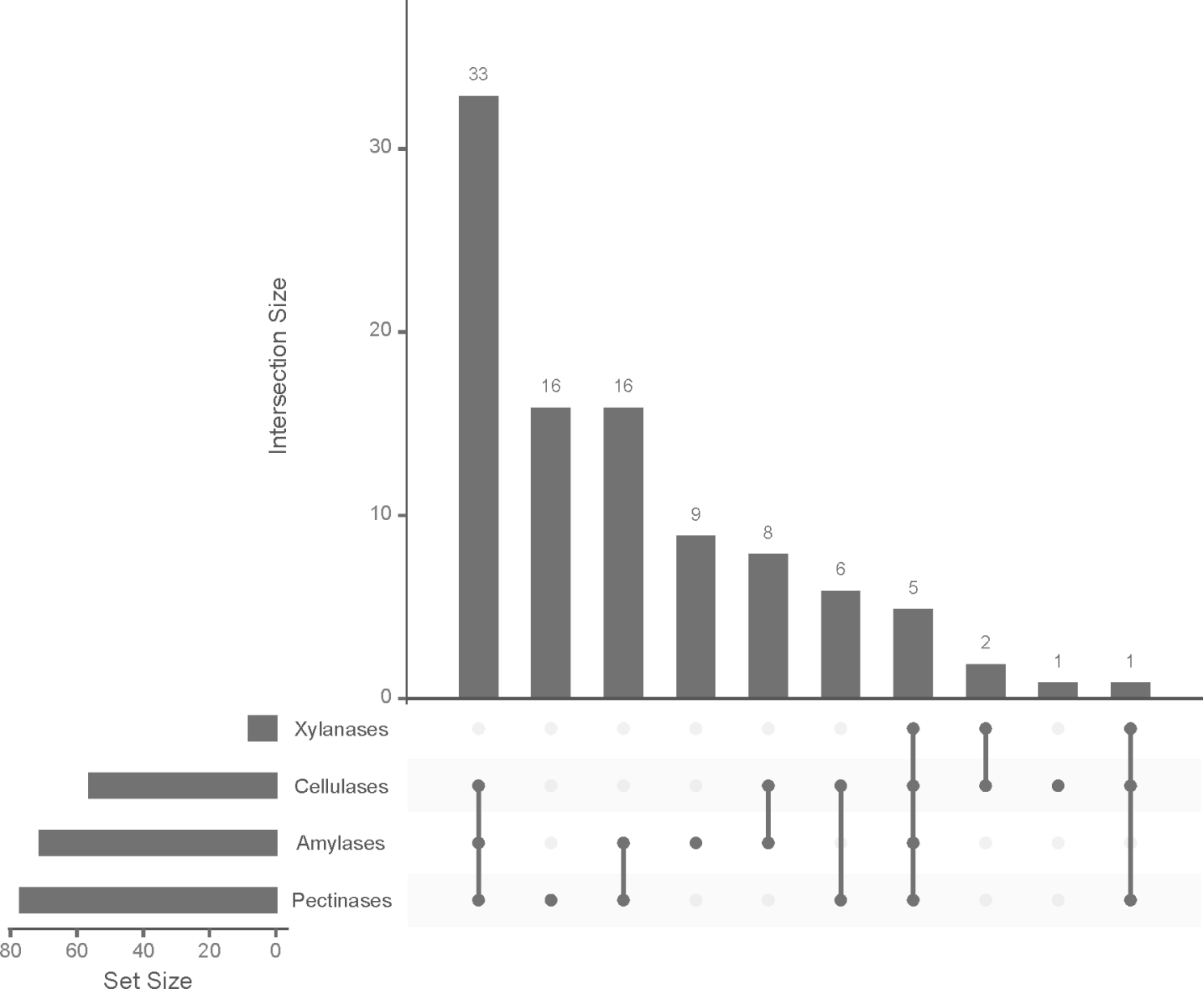
UpSet Graph representing the number of strains with solubilizing activity of one or more of the complex polysaccharides tested

Since *I. typographus* beetles cannot metabolize complex sugar molecules from the host tree, we tested the ability of the isolates to hydrolyse polysaccharides that can be found in the Spruce tree bark, which would suggest that the bacteriome might aid in the nutrition of the beetle. We found that almost 61% of the isolates showed in vitro pectinase activity (Figs. 4 and 5). None of the strains isolated from pupae showed this activity, whereas around 81% of strains isolated from adults and 59% of strains isolated from larvae hydrolysed pectin. Finally, almost 57% of the strains were able to hydrolyse starch, although the rate heightens to 83% in the adult group and is below 37% in the rest (Figs. 4 and 5).

On the other hand, only eight isolates hydrolysed xylan − *Curtobacterium flaccumfaciens* CYA5A, isolated from teneral adults, *Bacillus tequilensis* C3L6B, *Paenibacillus provencensis* C3L11A and C3L11B, isolated from larvae and *Pseudomonas typographi* CA3A (adult), C2L11, C2L12B, and C2L12C (larvae)−, but around 45% of the isolates had in vitro cellulolytic activity (Figs. 4 and 5), most of them adults (≈ 64% within this group) including *Erwinia* strains originating from all life stages and *Bacillus* strains coming from adults and larvae.

**Fig. 6:**
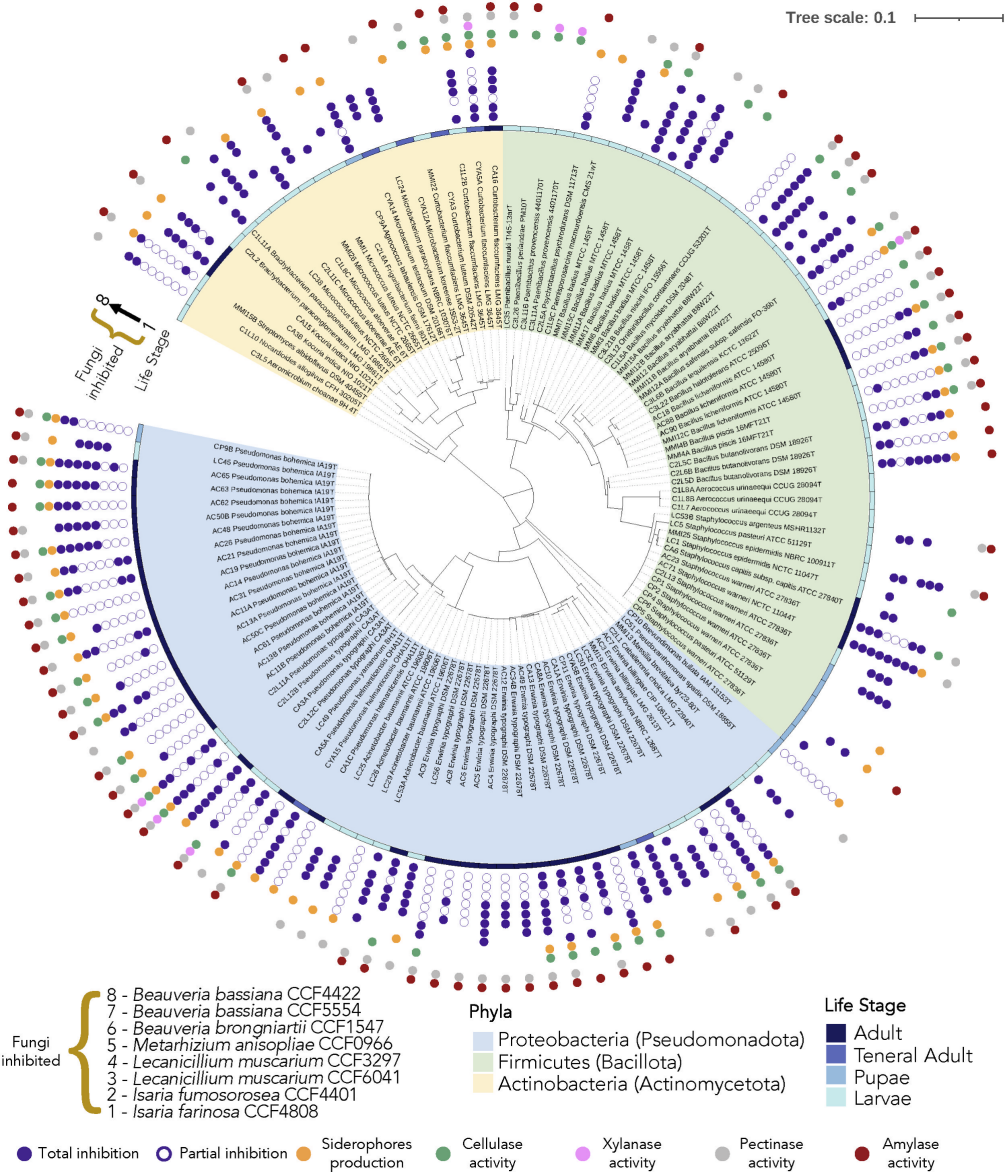
Phylogenetic tree constructed from isolate’s 16 S rRNA sequences. Dots indicate In vitro metabolic capacities. If dots are filled up in purple, total inhibition, if dots are not filled, strong inhibition against *Beauveria bassiana* CCF4422 (1), *Beauveria bassiana* CCF5554 (2), *Beauveria brongniartii* CCF1547 (3), *Metarhizium anisopliae* CCF0966 (4), *Lecanicillium muscarium* CCF3297 (5), *Lecanicillium muscarium* CCF6041 (6), *Isaria fumosorosea* CCF4401 (7), and *Isaria farinosa* CCF4808 (8), respectively from inside to outside the circumference. Dots coloured differently and rectangles indicate other features, as indicated in the legend

Out of all the isolates, strains C3L6B, CA3A, C2L11, C2L12B, and C2L12C were able to hydrolyse cellulose, xylan, starch, and pectin. Furthermore, thirty-two strains belonging to the genera *Erwinia, Bacillus, Pseudomonas, Paenibacillus*, and *Curtobacterium* showed cellulase, pectinase, and amylase activity. Also, a strain identified as *C. flaccumfaciens*, isolated from a teneral adult, showed cellulase, pectinase, and xylanase activities (Figs. 5 and 6).

### Antifungal capacity

Multiple biotic and abiotic threats menace *I. typographus* beetles during their life cycle, with entomopathogenic fungi being one of them [1, 9]. Thus, we evaluated the potential of the associated bacteriome to antagonize these fungi.

Firstly, we inoculated our strains on M9-CAS agar plates to study their capacity to produce siderophores, since these molecules are known for their antimicrobial capacity [46]. In total, 52.1% of our strains produced siderophores (Fig. 5). We observed that *Pseudomonas* and *Bacillus* strains isolated from larvae, teneral adults, and adults produced siderophores. Also, *Erwinia* strains from larvae, pupae, and adults, and *Curtobacterium* strains from larvae, teneral adults, and adults. Only *Staphylococcus* strains isolated from adults and pupae produced siderophores. We also observed that strains belonging to *E. typographi* and *P. bohemica* species, which were isolated from larvae, pupae, and adult phases, produced these molecules. This is also the case for *P. typographi* and *Bacillus licheniformis* strains isolated from larvae and adults (Fig. 6).

Secondly, we carried out an in vitro antifungal assay to evaluate the capacity of our isolates to produce metabolites that antagonize entomopathogenic fungi. We used 8 different *Ips* entomopathogenic strains belonging to the genera *Lecanicillium, Beauveria, Metarhizium*, and *Isaria.* Our results (Fig. 5, Supplementary Fig. 3) showed that only 5 strains, all isolated from larvae, exhibited no antagonistic activity to any of these fungi: C1L8A and C1L8B (*Aerococcus*), C3L21B (*Bacillus*), C3L26 (*Paenibacillus*), and MMI22 (*Curtobacterium*). Meanwhile, 95 strains were antagonistic to these fungi, being strains belonging to the genera *Erwinia, Pseudomonas, Brevundimonas, Bacillus, Nocardioides, Staphylococcus, Acinetobacter*, and *Streptomyces*. Of these, 37 strains obtained from larvae (13), pupae (1), teneral adult (1) and adults (21) strongly inhibited all fungi, including strains CA1A, CA8A, AC4, AC5, AC6, and AC8 (*E. typographi*), AC3(*E. billingiae*), and MMI19 (*E. amylovora*), which might belong to potentially new taxa included in the *Erwiniaceae* group. Other isolates with strong inhibitory capacities were AC7 (*E. amylovora*), CP9B, AC19, AC48, AC50B, AC61, AC62, and AC63 (*P. bohemica*), CA1C, CA5A, and CYA15 (*P. helmanticensis*), CP10 (*Brevundimonas bullata*), C3L6B (*B. tequilensis*), C3L22 (*Bacillus halotolerans* ATCC 25,096^T^), C2L5C, C2L5D, and C2L6B (*Bacillus butanolivorans*), C1L5A (*Bacillus mycoides*), MMI4A and MMI4B (*Bacillus piscis*), MMI6 (*Bacillus badius* MTCC), C1L10 (*Nocardioides allogilvus*), AC18, AC88, AC90, and MMI12C (*Bacillus licheniformis*), AC29 (*Acinetobacter baumannii*), AC71 (*S. warneri*), and MMI15B (*Streptomyces albidoflavus*).

## Discussion

The *I. typographus* beetles are devastating Norway Spruce woodlands in Europe due of population outbreaks caused by the climate change [1, 48]. Some authors have proposed that it’s bacteriome harbours ecologically important metabolic capacities potentially beneficial to the insect throughout its life cycle, which in turn could help it to overcome a hostile environment [1, 11, 12, 14]. Nevertheless, only a few studies exist that have embarked upon the composition of the bacteriome of the *I. typographus* bark beetle and its ecological role. Here we analyse the diversity of the bacteriome associated to the beetle during its different life stages by comparing the sequences of isolates and metabarcoding identifications. In addition, through in vitro assays, we have examined the metabolic capacities of the isolated strains and their potential roles in the host’s ecology.

Concerning the culture-independent analyses, it is reasonable to consider that the core bacteriome of *I. typographus* is highly dominated by the phylum Proteobacteria. We found families *Xanthomonadaceae* and *Erwiniaceae* to be predominant. These results agree with those presented by previous studies [17–19]. Although, in the metabarcoding analyses performed by Chakraborty et al. (Rouchovany, Czech Republic) [17] and Fang et al. (Jingouling Forest Farm, China) [18] hypervariable region V3-V4 was used instead of V5-V6, which was selected for our study and that of Veselská T. et al. (Protected Landscape Area Křivoklátsko, Czech Republic) [19] which can cause biases in the results [49]. Additionally, a putative undescribed genus from the *Enterobacterales* order is also present in all life stages, which was also found in other studies [18, 19]. Although Fang et al. [18] did not study teneral adults, and they split male adults from females. Nevertheless, they also observed that *Erwinia* were abundant in larvae and diminished at pupal stage, vice versa occurs with *Pseudoxanthomonas.* Chakraborty et al. [17] results showed *Rahnella* and *Raoultella* genera to be among the most abundant, these two were not found in our amplicon sequences, and the potentially novel *Enterobacterales* genus was not found by them. Nevertheless, *Pseudomonas* [19], *Acinetobacter*, and *Streptococcus* genera were also found in these studies [17, 18].

In our collection of isolates, strains belonging to *Pseudomonas* and *Erwinia* were amongst the most abundant. Moreover, our results also indicate that these genera are present over the whole life cycle of the beetle. *Erwinia* strains have already been related with beneficial traits in other insect hosts, shortening maturity time or heightening the oviposition rates [50]. Meanwhile, *Pseudomonas* has been proposed as an important taxon in bark beetle insects with different potential functions within their ecology [51]. Most of these strains were identified as *P. bohemica, P. typographi*, and *E. typographi.* All of them were firstly isolated from *Ips* beetles, the last two specifically from *I. typographus* [21, 22, 27]. In the case of *Staphylococcus* and *Curtobacterium* taxa, these were present in larval and adult stages and pupae and teneral adults respectively. *Bacillus* isolates encompass the most abundant genus in larval samples and were also present in adults. Strains belonging to the *Micrococcus* genus, previously isolated from *Ips* insects, were also identified. In contrast to the information provided by previous literature, we did not find *Rahnella, Serratia*, or *Arthrococcus* strains [20, 52, 53].

Our results of the metabarcoding alpha diversity (Shannon indices) and the bacterial communities isolated indicated an evolution of the bacteriome along the different life stages. Roughly, in the larval stage we observed a high degree of diversity, which decreased in pupae and remained lower during the teneral adult phase, but then recovered in adults. This trend was differently or not observed by other authors [18, 19], but it was similar in the case of the bark beetle *Dendroctonus rhizophagus*, although in this case teneral adults were not assessed [11]. Probably, this evolution in the bacteriome composition is caused by the morphological transformations of the beetle metamorphosis, which change the environmental conditions (host) for the bacterial community. However, a few taxa seem to remain present, suggesting an ecological role for the beetle independently of the life stage. In this sense, it has been observed that most of the bacteria associated with *I. typographus* beetles are acquired from the phloem [19], but vertical transmission, from adults to descendants, of some taxa might also be possible, as seen in other insects [54].

Additionally, we explored the potential ecological role of these bacterial strains in connection with two relevant aspects of the beetle: access to non-digestible carbon sources and protection against fungal entomopathogens. For some isolates, the capacity to hydrolyse CMC, xylan, pectin, and starch (all of them present in the inner bark) was promising. *B. tequilensis* and *P. typographi* strains originating from larvae and adults were able to strongly hydrolyse all polysaccharides. Another thirty-two strains, included into the genera *Erwinia*, *Bacillus*, *Pseudomonas*, *Paenibacillus*, and *Curtobacterium*, were capable of strongly hydrolyse 3 out of 4 of the polysaccharides tested (all but xylan). These results agree with those obtained for other bacterial strains isolated from *Ips* beetles that have been shown to synthesize enzymes with the capacity to hydrolyse these complex molecules into simpler sugars [20, 21]. Bark excavation is performed by the beetles during their mature (egg gallery system) and larval (galleries excavation) stages, which coincides with the stages in which the strains with the strongest hydrolytic capacities were obtained. Furthermore, according to our results bacteria associated to adults are more likely to show these activities. On the other side, in the massive amplicon analysis we found that a putative *Chitinophagacea* new genus was exclusively present in teneral adults. This family includes genera related with the hydrolyzation of chitin, as that of fungi and beetles [55, 56]. On one side, in this stage the beetle cuticle is not fully developed and starts to grow thicker [57], on the other it has also been observed that fungi sporulates in the pupal chambers, and when teneral adult feed they acquire them [58], what concurs with Veselská et al. [19] observations: they found the lowest fungi ASVs abundance in pupae samples, but it increased in teneral adults. These might explain why this taxon appears at this life stage.

Also, our findings indicate that strains belonging to the *Erwiniaceae* group and *Pseudomonas, Bacillus, Staphylococcus, Streptomyces, Acinetobacter*, and *Brevundimonas* genera can produce siderophores, these molecules can help out-compete microbes by reducing available iron [46]. Furthermore, the first four genera have already been reported to produce siderophores with antifungal activity [47, 59–62]. Strains belonging to the previous genera along with a *Nocardiodes* strain exhibited strong antifungal activity against 8 different strains of entomopathogenic fungi, indicating the production of bioactive compounds with antifungal activity. These genera have already been reported to synthesize molecules with this activity [63–70] such as a non-polyene antifungal extracted and purified from an *S. albidoflavus* strain [71]. The strongest inhibitory bacteria were isolated from larvae, which is reasonable considering it is in the larval stage when beetles are most susceptible to the attack of entomopathogenic fungi [72]. Although, in the adult phase we found the highest rate of strains with strong fungi inhibitory capacity.

## Conclusion

Our results indicate that isolates within the bacteriome of *I. typographus* beetle have the metabolic potential to produce different lytic enzymes that hydrolyse complex polysaccharides present in the wood, such as cellulose, pectin, starch and/or xylan, into simpler, assimilable forms for the beetle, potentially providing an additional source of carbon. Also, 83.9% of these isolates showed strong in vitro capacities to antagonize fungi entomopathogens, suggesting a protective role for the bacteriome. According to our results, bacteria with these capacities seem to be enriched in the larvae and adult phase. In the other hand, our taxonomical analysis shows that *E. typographi*, *P. bohemica*, and *P. typographi* species are repeatedly present within the bacteriome of *I. typographus* beetles, indicating that these species might be part of the core microbiome. These taxa showed strong capacity to produce lytic enzymes and antifungal compounds. These metabolic capacities were also observed in isolates included into the *Erwiniaceae* group, which according to our phylogenetic analysis, might belong to undescribed taxa that was also found in the metabarcoding analysis. Our results also suggest that adult beetles were more likely associated to bacteria that harbour these metabolic capacities, but bacteria obtained from larvae showed strongest inhibitory capacities. Also, strains included in *Staphylococcus*, *Acinetobacter*, *Curtobacterium, Streptomyces*, and *Bacillus* genera have the former metabolic capacities but are present in a lower frequency. Future studies involving bacterial-insect interactions would provide more insights into the bacteriome capacity to be beneficial to the beetle.

## Electronic supplementary material

Below is the link to the electronic supplementary material.

Supplementary Material 1

Supplementary Material 2

Supplementary Material 3

Supplementary Material 4

Supplementary Material 5

Supplementary Material 6

## References

[1] García-Fraile P (2018). Roles of bacteria in the bark beetle holobiont – how do they shape this forest pest?. Ann Appl Biol.

[2] Vitali V, Büntgen U, Bauhus J (2018). Seasonality matters—the effects of past and projected seasonal climate change on the growth of native and exotic conifer species in Central Europe. Dendrochronologia.

[3] Schebeck M, Hansen EM, Schopf A, Ragland GJ, Stauffer C, Bentz BJ (2017). Diapause and overwintering of two spruce bark beetle species. Physiol Entomol.

[4] Biedermann PHW, Müller J, Grégoire JC, Gruppe A, Hagge J, Hammerbacher A (2019). Bark Beetle Population Dynamics in the Anthropocene: Challenges and Solutions. Trends Ecol Evol.

[5] Wood TG, Thomas RJ. The mutualistic association between Macrotermitinae and Termitomyces. Insect-fungus interactions. 1989;14:69–92. 10.1016/C2009-0-02797-4.

[6] Mattanovich J, Ehrenhöfer M, Schafellner C, Tausz M, Führer E (2001). The role of sulphur compounds for breeding success of Ips typographus L. (Col., Scolytidae) on Norway Spruce (Picea abies [L.] Karst). J Appl Entomol.

[7] Morales-Jiménez J, Vera-Ponce de León A, García-Domínguez A, Martínez-Romero E, Zúñiga G, Hernández-Rodríguez C (2013). Nitrogen-Fixing and uricolytic Bacteria Associated with the gut of Dendroctonus rhizophagus and Dendroctonus valens (Curculionidae: Scolytinae). Microb Ecol.

[8] Wegensteiner R, Weiser J (2004). Annual variation of pathogen occurrence and pathogen prevalence in Ips typographus (Coleoptera, Scolytidae) from the BOKU University Forest Demonstration Centre. J Pest Sci.

[9] Wegensteiner R, Wermelinger B, Herrmann M (2015). Natural enemies of Bark Beetles: Predators, Parasitoids, Pathogens, and nematodes. In Bark Beetles: Biology and Ecology of native and invasive species. Elsevier sci.

[10] Wermelinger B (2004). Ecology and management of the spruce bark beetle Ips typographus—a review of recent research. For Ecol Manag.

[11] Morales-Jiménez J, Zúñiga G, Ramírez-Saad HC, Hernández-Rodríguez C (2012). Gut-Associated Bacteria throughout the life cycle of the Bark Beetle Dendroctonus rhizophagus Thomas and Bright (Curculionidae: Scolytinae) and their cellulolytic activities. Microb Ecol.

[12] Boone CK, Keefover-Ring K, Mapes AC, Adams AS, Bohlmann J, Raffa KF (2013). Bacteria Associated with a tree-killing insect reduce concentrations of Plant Defense Compounds. J Chem Ecol.

[13] Six DL (2013). The bark beetle holobiont: why microbes matter. J Chem Ecol.

[14] Cheng C, Wickham JD, Chen L, Xu D, Lu M, Sun J (2018). Bacterial microbiota protect an invasive bark beetle from a pine defensive compound. Microbiome.

[15] Six DL (2003). Bark beetle-fungus symbioses. Insect symbiosis.

[16] Chakraborty A, Modlinger R, Ashraf MZ, Synek J, Schlyter F, Roy A (2020). Core Mycobiome and their ecological relevance in the gut of five Ips bark beetles (Coleoptera: Curculionidae: Scolytinae). Front Microbiol.

[17] Chakraborty A, Ashraf MZ, Modlinger R, Synek J, Schlyter F, Roy A (2020). Unravelling the gut bacteriome of Ips (Coleoptera: Curculionidae: Scolytinae): identifying core bacterial assemblage and their ecological relevance. Sci Rep.

[18] Fang JX, Zhang SF, Liu F, Zhang X, Zhang FB, Guo XB (2020). Differences in gut bacterial Communities of Ips typographus (Coleoptera: Curculionidae) Induced by Enantiomer-Specific α-Pinene. Environ Entomol.

[19] Veselská T, Švec K, Kostovčík M, Peral-Aranega E, Garcia-Fraile P, Křížková B et al. Proportions of taxa belonging to the gut core microbiome change throughout the life cycle and season of the bark beetle Ips typographus. FEMS Microbiol Ecol. Under review. 10.1093/femsec/fiad072 37370225

[20] Fabryová A, Kostovčík M, Díez-Méndez A, Jiménez-Gómez A, Celador-Lera L, Saati-Santamaría Z (2018). On the bright side of a forest pest-the metabolic potential of bark beetles’ bacterial associates. Sci Total Environ.

[21] Peral-Aranega E, Saati-Santamaría Z, Kolařik M, Rivas R, García-Fraile P (2020). Bacteria belonging to pseudomonas typographi sp. Nov. from the bark beetle ips typographus have genomic potential to aid in the host ecology. Insects.

[22] Skrodenytee-Arbaciauskiene V, Radziute S, Stunzenas V, Buda V (2012). Erwinia typographi sp. nov., isolated from bark beetle (Ips typographus) gut. IJSEM.

[23] Cain CC, Henry AT, Waldo RH, Casida J, Falkinham JO (2000). Identification and characteristics of a novel Burkholderia strain with broad-spectrum antimicrobial activity. Appl Environ Microbiol.

[24] Raaijmakers JM, de Bruijn I, Nybroe O, Ongena M (2010). Natural functions of lipopeptides from Bacillus and Pseudomonas: more than surfactants and antibiotics. FEMS Microbiol Rev.

[25] Winding A, Binnerup SJ, Pritchard H (2004). Non-target effects of bacterial biological control agents suppressing root pathogenic fungi. FEMS Microbiol Ecol.

[26] González-Dominici LI, Saati-Santamaría Z, García-Fraile P (2021). Genome analysis and genomic comparison of the Novel Species Arthrobacter ipsi Reveal its potential protective role in its Bark Beetle host. Microb Ecol.

[27] Saati-Santamaría Z, López-Mondéjar R, Jiménez-Gómez A, Díez-Méndez A, Vetrovský T, Igual JM (2018). Discovery of phloeophagus beetles as a source of pseudomonas strains that produce potentially new bioactive substances and description of pseudomonas bohemica sp. nov. Front Microbiol.

[28] Rivas R, García-Fraile P, Mateos PF, Martínez-Molina E, Velázquez E (2007). Characterization of xylanolytic bacteria present in the bract phyllosphere of the date palm Phoenix dactylifera. Lett Appl Microbiol.

[29] Hall TA (1999). BioEdit: a user-friendly biological sequence alignment editor and analysis program for Windows 95/98/NT. Nucleic Acids Symp Ser.

[30] Kim OS, Cho YJ, Lee K, Yoon SH, Kim M, Na H (2012). Introducing EzTaxon-e: a prokaryotic 16s rRNA gene sequence database with phylotypes that represent uncultured species. IJSEM.

[31] Sagova-Mareckova M, Cermak L, Novotna J, Plhackova K, Forstova J, Kopecky J (2008). Innovative methods for soil DNA purification tested in soils with widely differing characteristics. Appl Environ Microbiol.

[32] Chelius MK, Triplett EW. The diversity of Archaea and Bacteria in Association with the roots of Zea mays L. Microb Ecol. 2001;252–63. 10.1007/s002480000087. 10.1007/s002480000087 11391463

[33] Redford AJ, Bowers RM, Knight R, Linhart Y, Fierer N (2010). The ecology of the phyllosphere: geographic and phylogenetic variability in the distribution of bacteria on tree leaves. Environ Microbiol.

[34] Minard G, Tran F-H, Dubost A, Tran-Van V, Mavingui P (2014). Pyrosequencing 16S rRNA genes of bacteria associated with wild tiger mosquito Aedes albopictus: a pilot study. Front Cell Infect Microbiol.

[35] Bolyen E, Rideout JR, Dillon MR, Bokulich NA, Abnet CC, Al-Ghalith GA (2019). Reproducible, interactive, scalable and extensible microbiome data science using QIIME 2. Nat. Biotechnol.

[36] Callahan BJ, Sankaran K, Fukuyama JA, McMurdie PJ, Holmes SP. Bioconductor workflow for microbiome data analysis: from raw reads to community analyses. F1000research. 2016;5. https://doi.org/10.12688%2Ff1000research.8986.2. 10.12688/f1000research.8986.1 PMC4955027 27508062

[37] Bokulich NA, Kaehler BD, Rideout JR, Dillon M, Bolyen E, Knight R (2018). Optimizing taxonomic classification of marker-gene amplicon sequences with QIIME 2’s q2-feature-classifier plugin. Microbiome.

[38] Kumar S, Stecher G, Li M, Knyaz C, Tamura K (2018). MEGA X: Molecular Evolutionary Genetics Analysis across Computing Platforms. Mol Biol Evol.

[39] Thompson JD, Higgins DG, Gibson TJ, CLUSTAL W (1994). Improving the sensitivity of progressive multiple sequence alignment through sequence weighting, position-specific gap penalties and weight matrix choice. Nucleic Acids Res.

[40] Larkin MA, Blackshields G, Brown NP, Chenna R, Mcgettigan PA, McWilliam H (2007). Clustal W and Clustal X version 2.0. Bioinformatics.

[41] Tamura K, Nei M (1993). Estimation of the number of nucleotide substitutions in the control region of mitochondrial DNA in humans and chimpanzees. Mol Biol Evol.

[42] Mateos PF, Jimenez-Zurdo JI, Chen J, Squartini AS, Haack SK, Martinez-Molina E (1992). Cell-associated pectinolytic and cellulolytic enzymes in Rhizobium leguminosarum biovar trifolii. App Environ Microbiol.

[43] García-Fraile P, Rivas R, Willems A, Peix A, Martens M, Martínez-Molina E (2007). Rhizobium cellulosilyticum sp. nov., isolated from sawdust of Populus alba. IJSEM.

[44] Jiménez-Gómez A, Saati-Santamaría Z, Igual JM, Rivas R, Mateos PF, García-Fraile P (2019). Genome insights into the Novel Species Microvirga brassicacearum, a rapeseed endophyte with biotechnological potential. Microorganisms.

[45] Kubátová A, Dvořák L (2005). Entomopathogenic fungi associated with insect hibernating in underground shelters. Czech Mycol.

[46] Pažoutová S, Šrůtka P, Holuša J, Chudíčková M, Kolařík M (2010). Diversity of xylariaceous symbionts in Xiphydria woodwasps: role of vector and a host tree. Fungal Ecol.

[47] Sulochana MB, Jayachandra SY, Kumar SKA, Dayanand A (2014). Antifungal attributes of siderophore produced by the Pseudomonas aeruginosa JAS-25. J Basic Microbiol.

[48] Biedermann PHW, Müller J, Grégoire JC, Gruppe A, Hagge J, Hammerbacher A (2019). Bark Beetle Population Dynamics in the Anthropocene: Challenges and Solutions. Trends Ecol Evol.

[49] Claesson MJ, Wang Q, O’Sullivan O, Greene-Diniz R, Cole JR, Ross RP, O’Toole PW (2010). Comparison of two next-generation sequencing technologies for resolving highly complex microbiota composition using tandem variable 16S rRNA gene regions. Nucleic Acids Res.

[50] De Vries EJ, Jacobs G, Sabelis MW, Menken SB, Breeuwer JA. Diet–dependent effects of gut bacteria on their insect host: the symbiosis of Erwinia sp. and western flower thrips. Proc. Royal Soc. B, 2004;271(1553):2171–2178. 10.1098/rspb.2004.2817. 10.1098/rspb.2004.2817 PMC1691834 15475338

[51] Saati-Santamaría Z, Rivas R, Kolařik M, García-Fraile P (2021). A new perspective of Pseudomonas—host interactions: distribution and potential ecological functions of the genus Pseudomonas within the Bark Beetle Holobiont. Biology.

[52] Yilmax H, Sezen K, Kati H, Demirbaǧ Z (2006). The first study on the bacterial flora of the European spruce bark beetle, Dendroctonus micans (Coleoptera: Scolytidae). Biología.

[53] Hu X, Yu J, Wang C, Chen H (2014). Cellulolytic Bacteria Associated with the gut of Dendroctonus armandi Larvae (Coleoptera: Curculionidae: Scolytinae). Forests.

[54] Bright M, Bulgheresi S (2010). A complex journey: transmission of microbial symbionts. Nat Rev Microbiol.

[55] Carter DO, Metcalf JL, Bibat A, Knight R (2015). Seasonal variation of postmortem microbial communities. Forensic Sci Med Pathol.

[56] Muthukrishnan S, Mun S, Noh MY, Geisbrecht ER, Arakane Y (2020). Insect cuticular chitin contributes to form and function. Curr Pharm Des.

[57] Banskar S, Mourya DT, Shouche YS (2016). Bacterial diversity indicates dietary overlap among bats of different feeding habits. Microbiol Res.

[58] Six DL (2012). Ecological and evolutionary determinants of bark beetle—fungus symbioses. Insects.

[59] Wilson MK, Abergel RJ, Raymond KN, Arceneaux JEL, Byers BR (2006). Siderophores of Bacillus anthracis, Bacillus cereus, and Bacillus thuringiensis. Biochem Biophys Res Commun.

[60] Yu X, Ai C, Xin L, Zhou G (2011). The siderophore-producing bacterium, Bacillus subtilis CAS15, has a biocontrol effect on Fusarium wilt and promotes the growth of pepper. Eur J Soil Biol.

[61] Raaska L, Mattila-Sandholm T (1995). Effects of iron level on the anatagonistic action of siderophores from non-pathogenic Staphylococcus spp. J Ind Microbiol Biotechnol.

[62] Nagpure A, Choudhary B, Kumar S, Gupta RK (2014). Isolation and characterization of chitinolytic Streptomyces sp. MT7 and its antagonism towards wood-rotting fungi. Ann Microbiol.

[63] El-Goorani MA, Hassanein F, Shoeib A (1992). Antibacterial and antifungal spectra of antibiotics produced by different strains of Erwinia herbicola (Pantoea agglomerans). J phytopathol.

[64] Tenning P, van Rijsbergen R, Zhao Y, Joos H (1993). Cloning and transfer of genes for antifungal compounds from Erwinia herbicola to Escherichia coli. Mol. Plant Microbe Interact.

[65] Liu CH, Chen X, Liu TT, Lian B, Gu Y, Caer V, Xue YR, Wang BT (2007). Study of the antifungal activity of Acinetobacter baumannii LCH001 in vitro and identification of its antifungal components. Appl Microbiol Biotech.

[66] Prapagdee B, Kuekulvong C, Mongkolsuk S (2008). Antifungal potential of extracellular metabolites produced by Streptomyces hygroscopicus against phytopathogenic fungi. Int J Biol Sci.

[67] León M, Yaryura PM, Montecchia MS, Hernández AI, Correa OS, Pucheu NL et al. Antifungal Activity of Selected Indigenous Pseudomonas and Bacillus from the Soybean Rhizosphere. Int J Microbiol. 2009;2009. 10.1155/2009/572049. 10.1155/2009/572049 PMC2789335 20016811

[68] Kupferschmied P, Maurhofer M, Keel C (2013). Promise for plant pest control: root-associated pseudomonads with insecticidal activities. Front Plant Sci.

[69] Bhattacharjee R (2014). An overview of fungal and bacterial biopesticides to control plant pathogens/diseases. Afr J Microbiol Res.

[70] Alijani Z, Amini J, Ashengroph M, Bahramnejad B (2019). Antifungal activity of volatile compounds produced by Staphylococcus sciuri strain MarR44 and its potential for the biocontrol of Colletotrichum nymphaeae, causal agent strawberry anthracnose. Int J Food Microbiol.

[71] Augustine SK, Bhavsar SP, Kapadnis BP (2005). A non-polyene antifungal antibiotic from Streptomyces albidoflavus PU 23. J Biosci.

[72] Erler F, Ates AO (2015). Potential of two entomopathogenic fungi, Beauveria bassiana and metarhizium anisopliae (Coleoptera: Scarabaeidae), as biological control agents against the June beetle. J Insect Sci.

