# Supplementary figures and images for: New insight into the bark beetle ips typographus bacteriome reveals unexplored diversity potentially beneficial to the host
Source: Environ Microbiome. 2023 Jun 9;18:53. doi: 10.1186/s40793-023-00510-z (PMC10257263; doi:10.1186/s40793-023-00510-z)

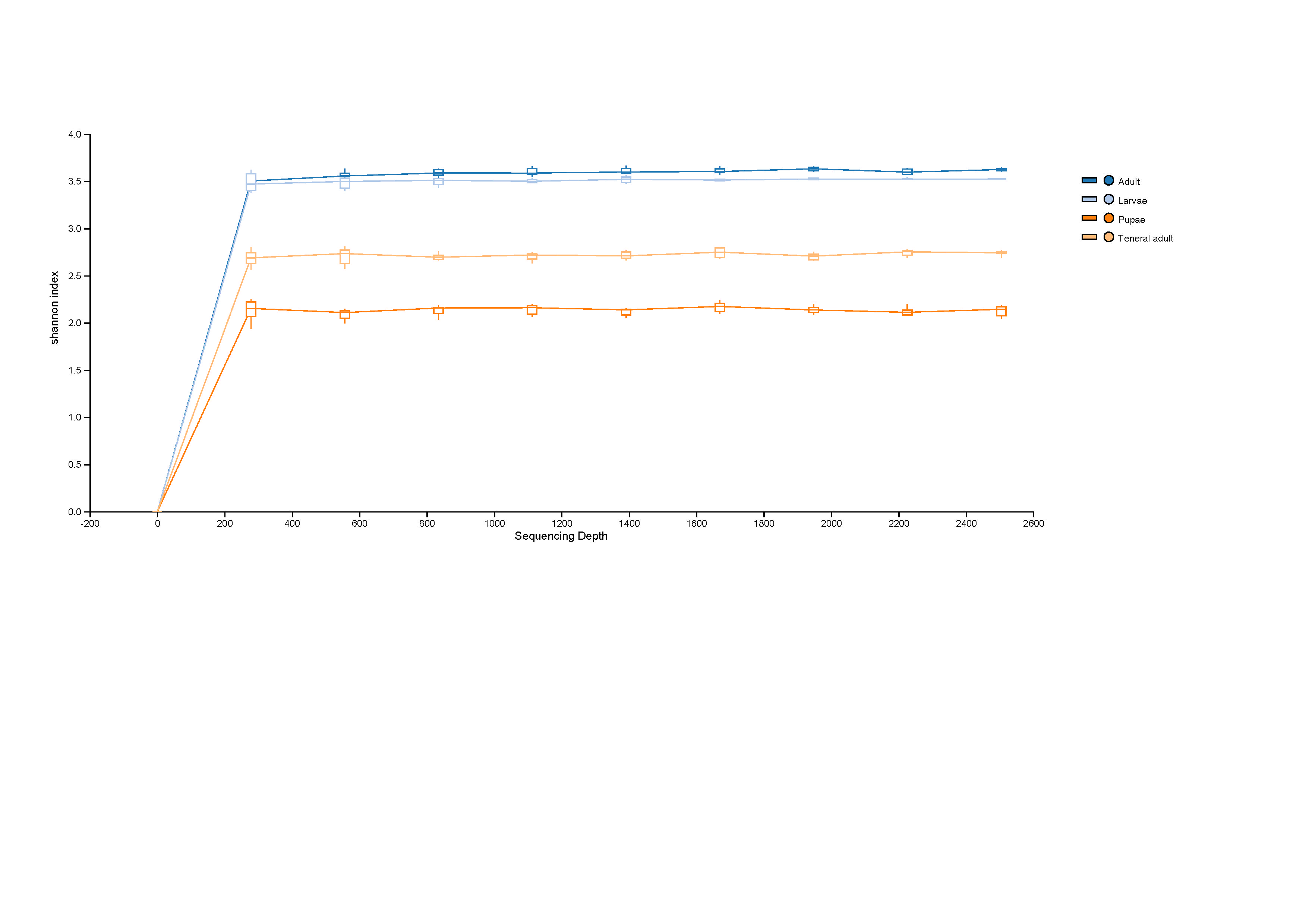

Supplement: Supplementary file 1 — Supplementary Material 1 [file 40793_2023_510_MOESM1_ESM.tif]

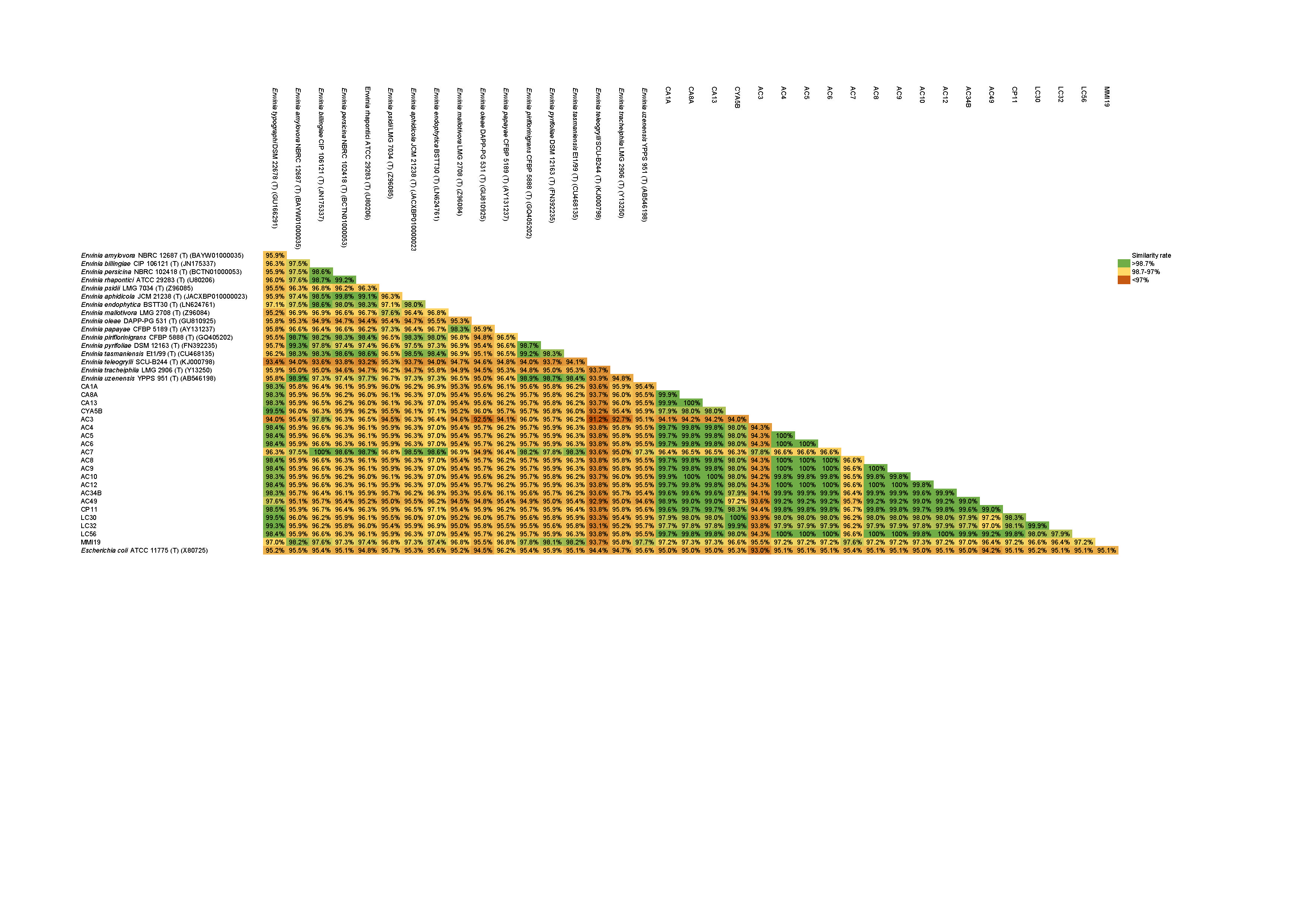

Supplement: Supplementary file 3 — Supplementary Material 3 [file 40793_2023_510_MOESM3_ESM.tif]

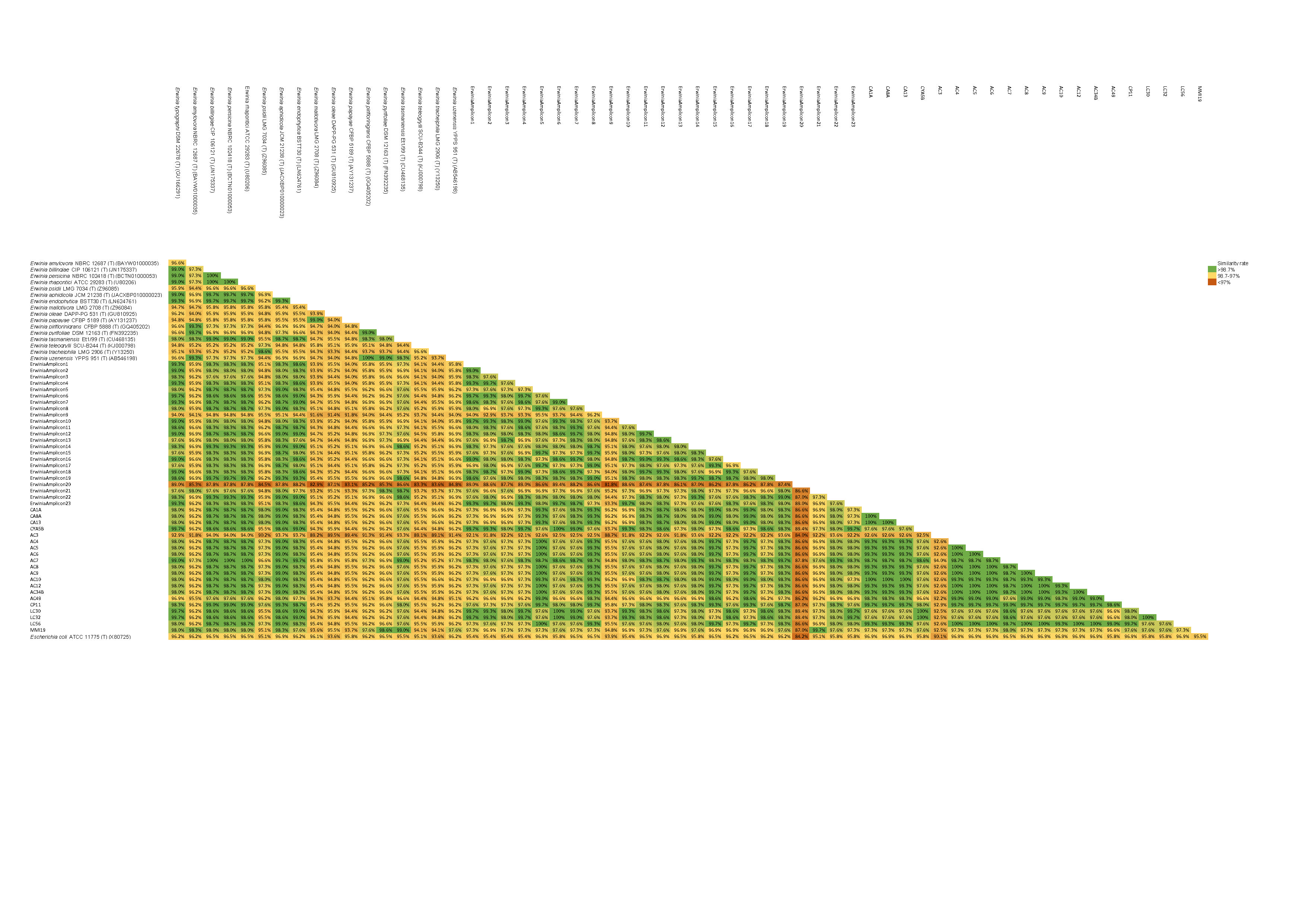

Supplement: Supplementary file 4 — Supplementary Material 4 [file 40793_2023_510_MOESM4_ESM.tif]

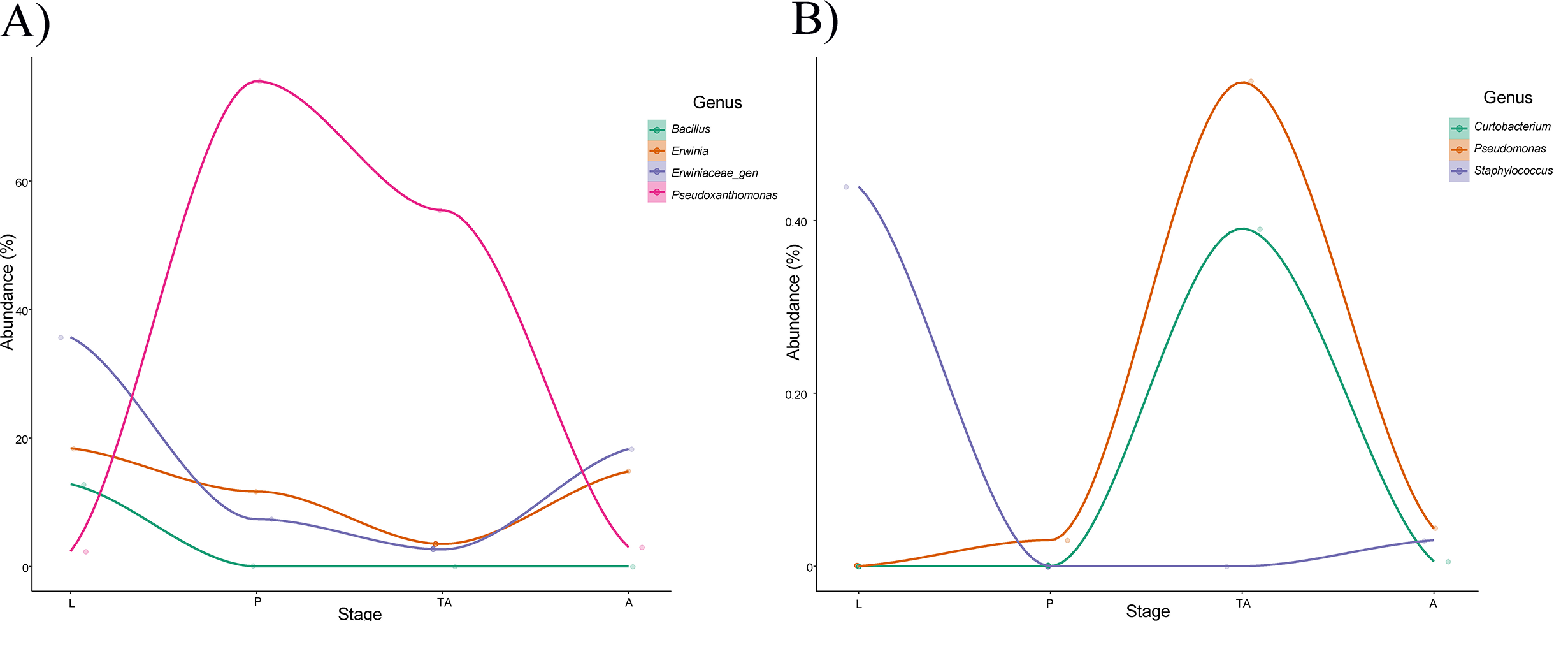

Supplement: Supplementary file 5 — Supplementary Material 5 [file 40793_2023_510_MOESM5_ESM.tif]

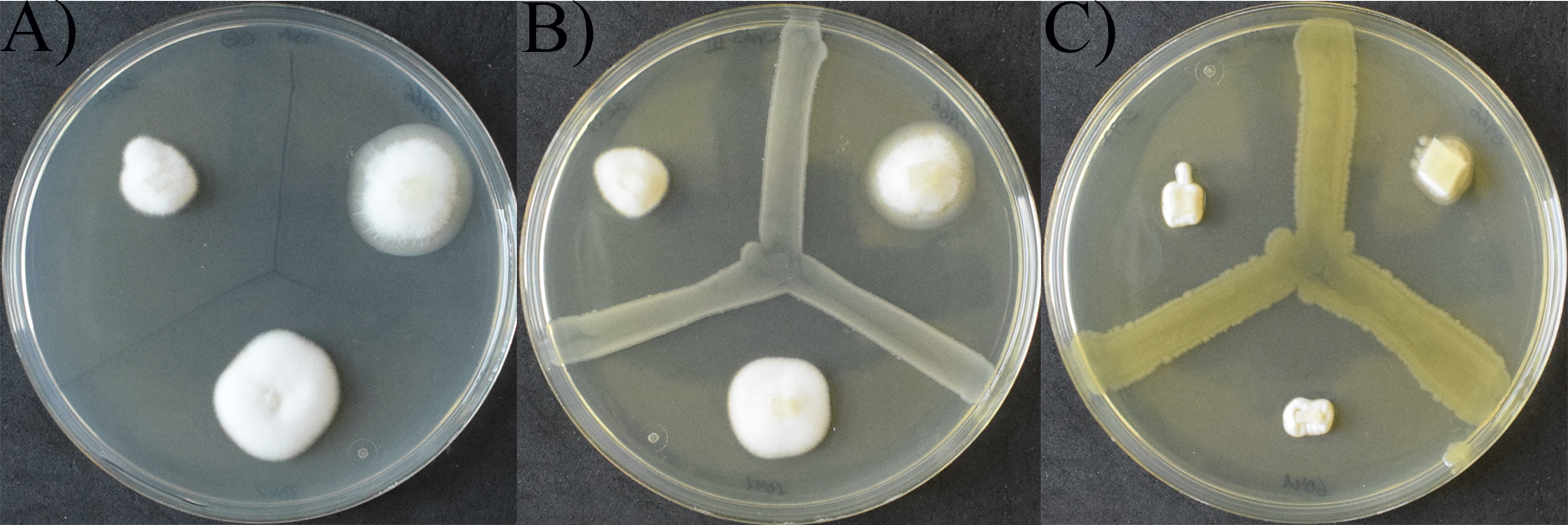

Supplement: Supplementary file 6 — Supplementary Material 6 [file 40793_2023_510_MOESM6_ESM.tif]
